# Toward a Computational Neuroethology of Vocal Communication: From Bioacoustics to Neurophysiology, Emerging Tools and Future Directions

**DOI:** 10.3389/fnbeh.2021.811737

**Published:** 2021-12-20

**Authors:** Tim Sainburg, Timothy Q. Gentner

**Affiliations:** ^1^Department of Psychology, University of California, San Diego, La Jolla, CA, United States; ^2^Center for Academic Research & Training in Anthropogeny, University of California, San Diego, La Jolla, CA, United States; ^3^Neurosciences Graduate Program, University of California, San Diego, La Jolla, CA, United States; ^4^Neurobiology Section, Division of Biological Sciences, University of California, San Diego, La Jolla, CA, United States; ^5^Kavli Institute for Brain and Mind, University of California, San Diego, La Jolla, CA, United States

**Keywords:** neuroethology, computational neuroethology, denoising, vocalization, UMAP, sequence model, morph

## Abstract

Recently developed methods in computational neuroethology have enabled increasingly detailed and comprehensive quantification of animal movements and behavioral kinematics. Vocal communication behavior is well poised for application of similar large-scale quantification methods in the service of physiological and ethological studies. This review describes emerging techniques that can be applied to acoustic and vocal communication signals with the goal of enabling study beyond a small number of model species. We review a range of modern computational methods for bioacoustics, signal processing, and brain-behavior mapping. Along with a discussion of recent advances and techniques, we include challenges and broader goals in establishing a framework for the computational neuroethology of vocal communication.

## 1. Introduction

Over the past several years emerging methods have enabled biologists to capture and quantify ethological data in ways that yield new insights into the structure and organization of behavior. These methods capitalize on two advances: the ability to record and annotate very-large behavioral datasets, and the use of new computational tools to reveal structure within and between these datasets. The ethological and neuro-ethological study of animal communication has a long history, and its future stands to benefit greatly from these new methods. Here, we discuss this emerging set of tools available to the animal communication researcher. We contextualize these computational methods within the emerging field of computational ethology more broadly and discuss how these tools can be applied in behavior and neurophysiology.

Many of the challenges that exist in the computational neuroethology of vocal behavior are neither new nor unique and parallel those in other areas of human and animal behavior. For example, the algorithmic discovery of vocal units and sequential organization in animal communication parallels the zero-speech challenge in language acquisition: given limited sensory information, can we build a system that discovers subwords, words, and sequential and syntactic organization present in speech (Versteegh et al., 2015). In animal communication the challenge is similar: can we infer vocal segment boundaries, categories, and temporal organization from the physical and temporal characteristics of the signal. The computational neuroethology of vocal communication also parallels the emerging field of motion sequencing and the mapping behavioral kinematics, where new technologies allowing researchers to map postures and behavioral kinematics have facilitated new understandings of behavioral dynamics across scales (Anderson and Perona, 2014; Berman, 2018; Brown and De Bivort, 2018; Christin et al., 2019; Datta et al., 2019; Pereira et al., 2019). It is the goal of computational neuroethology to not only develop an understanding of the organization of behaviors, but also the neural and cognitive mechanisms that facilitate behavior. This review synthesizes work from several fields including bioacoustics, systems neuroscience, and computational neuroethology to discuss emerging methodologies and frameworks which span these fields and are available to vocal communication researchers.

The review begins with considerations in bioacoustics and signal processing and then shifts to a consideration of acoustic structure, sequential organization, and eventually to mapping the acoustic and sequential structure of vocal communication to neurophysiology correlates of behavior and perception. Throughout our review of current approaches, we relay ongoing challenges, discuss future directions, and attempt to give practical advice on vocal analyses.

## 2. Signal Processing and Denoising

Recorded sounds typically contain a mixture of both relevant and irrelevant components. Computational ethology often relies on modeling structure in data without making assumptions about the relevant features. Thus it is often important to remove irrelevant features (i.e., background noise) prior to analysis. Ones operationalization of noise can vary based upon the end goal of the analysis. A simple example is band-pass filtering: because vocalizations typically occur in a confined frequency range, it is reasonable to consider signal outside of that range noise and filter it away. When a recording contains vocalizations from two animals, a songbird with song in a high-frequency range, and heterospecific calls in a low-frequency range, if the subject of interest is the songbird, a simple high-pass filter can be applied to attenuate the non-target calls. When frequency ranges overlap between signal and noise, however, the problem of noise reduction becomes more difficult.

### 2.1. Noise Reduction

**Figure 1:**
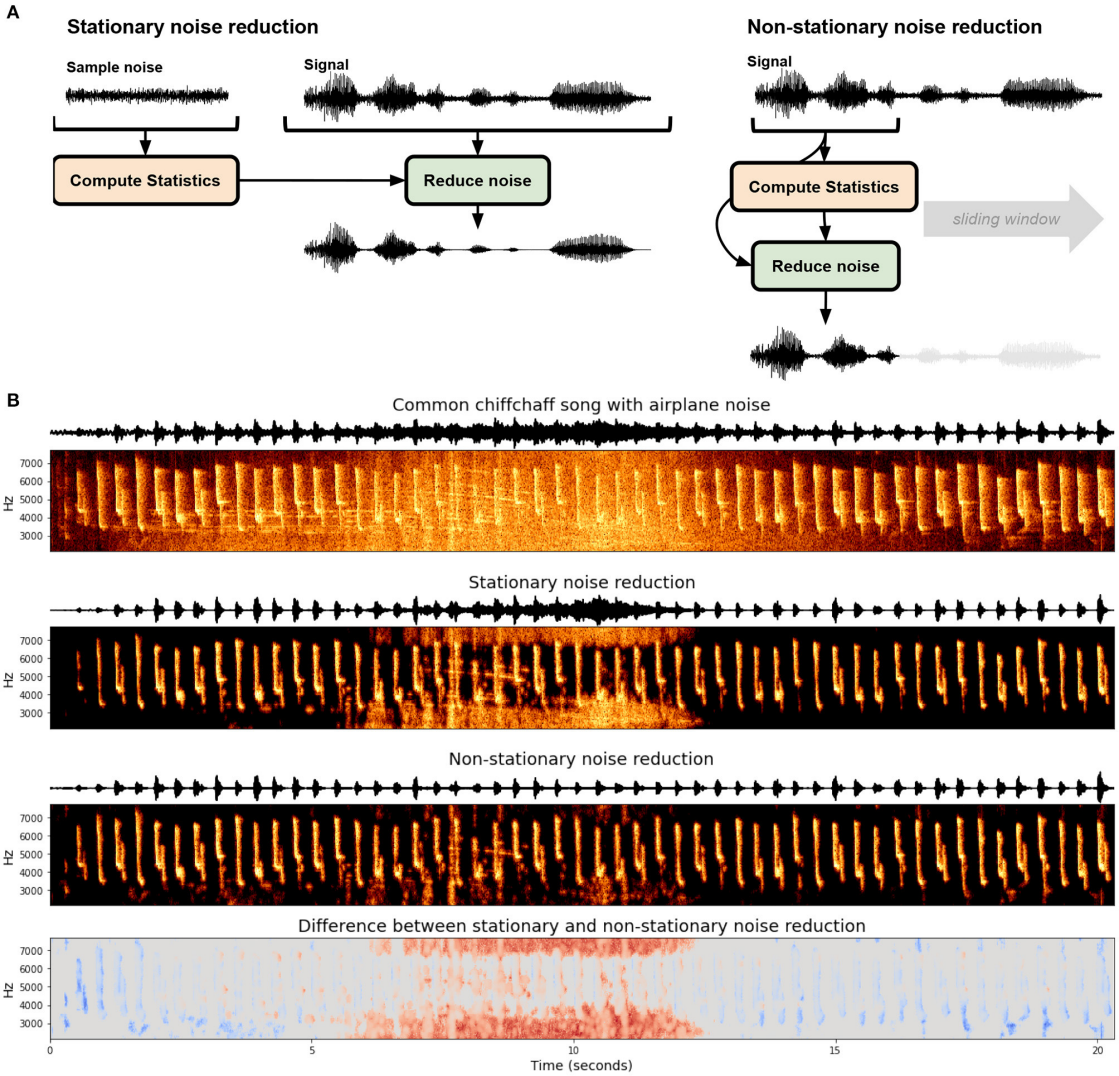
Stationary and non-stationary spectral gating noise reduction. **(A)** An overview of each algorithm. Stationary noise reduction typically takes in an explicit noise signal to calculate statistics and performs noise reduction over the entire signal uniformly. Non-stationary noise reduction dynamically estimates and reduces noise concurrently. **(B)** Stationary and non-stationary spectral gating noise reduction using the noisereduce Python package (Sainburg, 2019) applied to a Common chiffchaff (*Phylloscopus collybita*) song (Stowell et al., 2019) with an airplane noise in the background. The bottom frame depicts the difference between the two algorithms.

Determining what constitutes noise in recordings is non-trivial and impacts what type of noise reduction algorithm can and should be used. In a systematic review of noise reduction methods in bio-acoustics, Xie et al. (2020) outline six classes of noise reduction algorithms used for bio-acoustics: (1) Optimal FIR filter (e.g., Kim et al., 2000), (2) spectral subtraction (e.g., Boll, 1979; Kiapuchinski et al., 2012; Sainburg et al., 2020b), (3) minimum-mean square error short-time spectral amplitude estimator (MMSE-STSA; e.g., Ephraim and Malah, 1984; Alonso et al., 2017; Brown et al., 2017) (4) wavelet based denoising (e.g., Ren et al., 2008; Priyadarshani et al., 2016) (5) image processing based noise reduction, and (6) deep learning based noised reduction. These noise reduction algorithms can be broadly divided into two categories: stationary and non-stationary noise reduction (Figure 1A). Stationary noise reduction acts on noise that is stationary in intensity and spectral shape over time, such as the constant hum of electronics. Non-stationary noise reduction targets background noise that is non-stationary and can fluctuate in time, like the on-and-off presence of a plane flying overhead (Figure 1B). Stationary noise reduction algorithms operationalize noise as stationary signals, for example, the constant hum from a nearby electronic device in a laboratory setting, or insect noise in a field setting.

One approach to stationary noise reduction is spectral gating, a spectral-subtraction algorithm (e.g., Kiapuchinski et al., 2012; Sainburg et al., 2020b). The general notion is that for each frequency component of the signal, any time-frequency component below a threshold is discarded as noise. Spectral gating computes the mean and standard deviation of each frequency channel of a Short-Time Fourier Transform (STFT) of a signal (e.g., a spectrogram) and optionally a noise clip. A threshold, or gate, for each frequency component is then set at some level above the mean (e.g., three standard deviations). This threshold determines whether a time-frequency component in the spectrogram is considered signal or noise. The spectrogram is then masked based upon this threshold and inverted (with an inverse STFT) back into the time domain.

### 2.2. Non-stationary Noise Reduction

While stationary noise reduction algorithms can operationalize noise as any stationary acoustic signal, non-stationary algorithms vary in how they determine what is signal and what is noise. Non-stationary noise can be more challenging to remove because it can be difficult to algorithmically define the difference between signal and noise. Because the hum of a computer in the background of a lab-recording is stationary, it can be defined as noise and can be readily removed. A bird hopping around its cage can produce time-varying sounds in the same frequency range as song, making it especially pernicious.

One approach for determining the boundary between signal and non-stationary noise is to determine the timescale on which the signal acts and treat anything outside of that timescale as noise. For example, zebra finch motifs are generally between 0.5 and 1.5 s long repeated one to four times (Bruno and Tchernichovski, 2019). Any acoustic event that is outside of that time range could be considered noise. Spectral gating can be extended to non-stationary noise reduction by computing a variable gate based upon the current estimate of background noise. In the Python package noisereduce (Sainburg, 2019), this background estimate is computed using a time-smoothed spectrogram (using a forward and backward IIR filter) on a timescale parameterized by the expected signal length, an approach motivated by the Per-Channel Energy Normalization algorithm (outlined in Section 3). An example of this is given in Figure 1, where stationary and non-stationary spectral gating noise reduction is applied to birdsong with an airplane noise occurring in the background of the middle of the recording. Because the airplane noise is non-stationary, The stationary approach fails in two ways relative to the non-stationary approach: the airplane noise is not fully successfully gated at its peak in the middle of the recording (shown as red in the bottom panel) and weaker syllables of song are treated as noise and reduced in the beginning and end of the clip (shown in blue in the bottom panel). Advantages of non-stationary noise reduction are not unique to acoustic noise: when we know the timescale of a signal we can use the same non-stationary principles to remove noise occurring at different timescales. For example in the continuous recording of neural data, action potentials occur within the range of 1 ms. Events occurring over tens or hundreds of milliseconds can therefore be treated as noise.

### 2.3. Reducing Noise With Deep Learning

A promising future avenue for noise reduction is in explicitly training machine learning algorithms to mask or remove noise, as is done in speech enhancement and segregation (Wang and Chen, 2018). At present, however, deep learning based noise reduction has not been utilized directly in bio-acoustics (Xie et al., 2020). Xie et al. (2020) attribute this to a lack of utility when using denoising in some applications of deep learning-based bio-acoustics detection (Kong et al., 2017). The utility of noise reduction exists beyond classification tasks, however. For example, computing spectral features and acoustic similarity between vocalizations can be susceptible to background noise. Recent work by Stowell et al. (2019) shows that manipulating datasets by superimposing background environment noise on vocal datasets can reduce confounds and improve identification across recording conditions. Similar approaches could be used to remove noise. For example, spectral gating could be extended with neural networks by training a neural network to learn a mask to gate away background noise and recover the lower-noise spectrogram, as has been done in speech enhancement applications (Wang and Chen, 2018; Lee and Kim, 2020).

It is also important to consider what information is being removed by pre-processing techniques such as denoising. Pre-processing methods throw away potentially valuable information that will influence downstream analyses. De-noising vocal data without careful consideration can remove lower amplitude syllables of birdsong or infrequent vocalizations outside of the expected frequency range.

## 3. Signal Representation

An important consideration in any analysis pipeline is how to represent the data that goes in. Animal vocalizations are typically recorded using one or more microphones at a sampling rate that can capture the full spectral range of the vocalization. Performing analyses directly upon recorded waveforms is not always optimal for capturing informative structure in vocal data, however. Waveforms are high-dimensional representations of audio that can make it difficult for learning algorithms to capture time-frequency structure in vocalizations. Spectro-temporal representations can be both lower-dimensional, and more explicitly capture complex time-frequency relationships in vocalizations.

**Figure 2:**
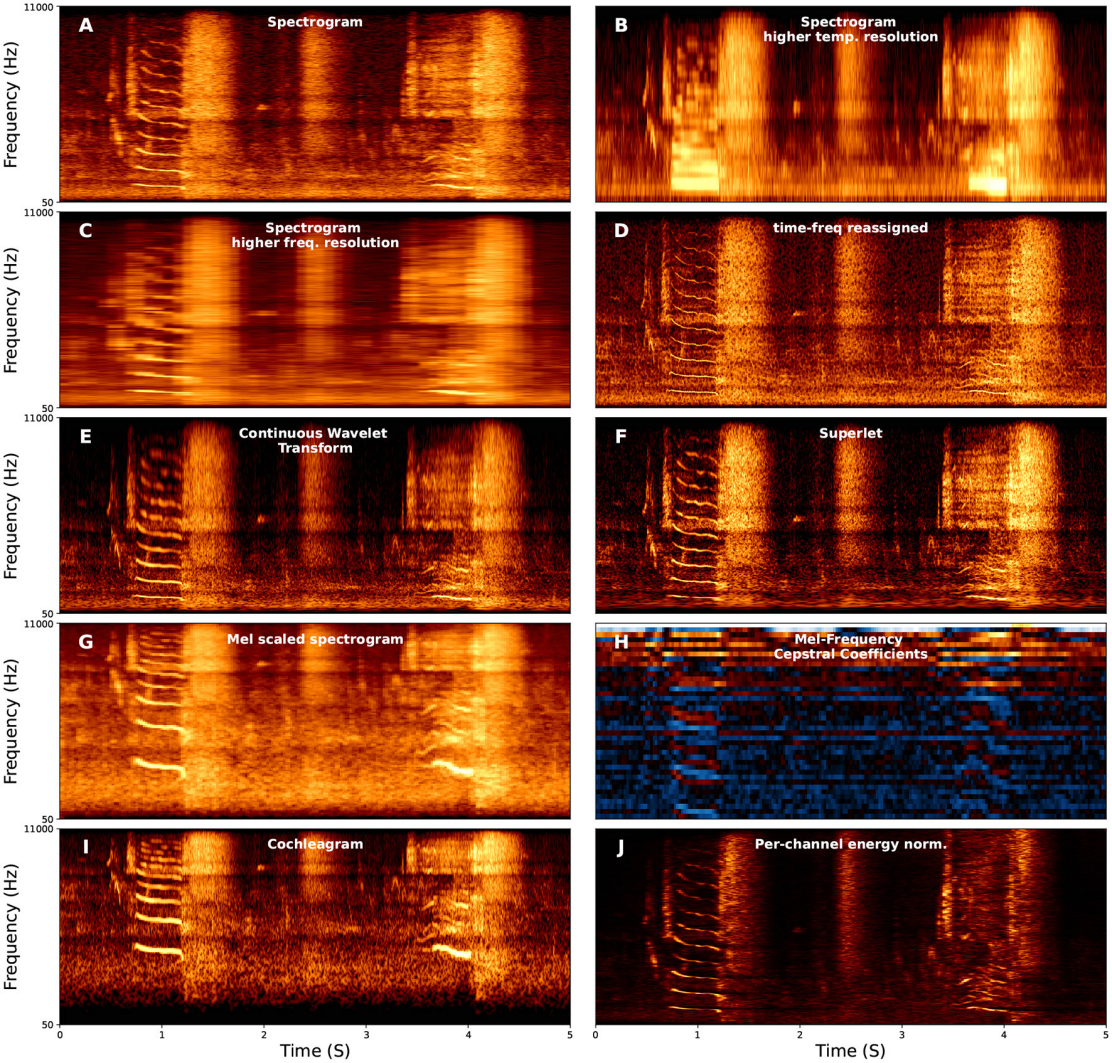
Examples of several different spectral representations of a five-second red deer (*Cervus elaphus*) vocalization. For each axis, the *x*-axis corresponds to time, and the *y*-axis corresponds to frequency. The y-axis corresponds to frequency and is linearly spaced in **(A–F,J)** between 50 and 11,000 Hz and log-spaced in the same range for **(G–I)**. **(F)** Continuous Wavelet Transform using the Morlet (i.e., Gabor) wavelet.

Spectrograms are, at present, the most common form of vocalization representation, both for visualization and as input to learning algorithms, both in bio-acoustics and speech. When representing vocal data with a spectrogram, the parameters used to compute the spectrogram can have an important influence on the performance of the algorithm (Elie and Theunissen, 2016; Knight et al., 2020). The most important parameterization of spectrograms is the trade-off between temporal and frequency resolution when computing a spectrogram, a result of the Heisenburg Uncertainty Principle (Gardner and Magnasco, 2006; Moca et al., 2021). For example, three spectrograms are shown in Figures 2A–C with different windows used to compute the Short-Time Fourier Transform. The first has an intermediate-sized window with intermediate time and frequency resolution (Figure 2A), the second uses a short window with high time-resolution and low frequency-resolution (Figure 2B), and the third uses a long window with high frequency-resolution but low time-resolution (Figure 2C).

A number of approaches exist to improve time and frequency resolution. Time-frequency reassigned spectrograms attempt to improve time-frequency resolution using additional information from the phase spectrum (Figure 2D) (Fulop and Fitz, 2006; Gardner and Magnasco, 2006; Xiao and Flandrin, 2007). Wavelet transforms (Figure 2E) have more recently been used in representing animal vocalizations (Main and Thornton, 2015; Priyadarshani et al., 2016, 2020; Hsu et al., 2018), and allows multi-scaled emphasis on time vs. frequency, for example emphasizing frequency resolution at lower frequencies and time-resolution at higher frequencies, intuitively because an uncertainty of 50 Hz is more relevant at 500 Hz than at 5,000 Hz. Most recently, the superlet (Figure 2F) enables time-frequency super-resolution by geometrically combining sets of wavelets with increasing constrained bandwidths (Moca et al., 2021).

There are also several variants of spectrograms and time-frequency representations that differentially emphasize time-frequency information. For example, log-scaling spectrograms in frequency emphasizes lower frequency ranges over higher frequency ranges, which parallels both the cochlea and perception (Eldredge et al., 1981). Mel-scaling (Figure 2G), is a form of log-scaling fit to fit human perception (Stevens et al., 1937), though the specific scaling range relative to human perception are imperfect (Greenwood, 1997). Mel-Frequency Cepstral Coefficients (MFCCs; Figure 2H) additionally compute the Discrete Cosine Transform on the Mel-spectrogram, and were, until recently, commonly used for speech recognition because they are generally robust to noise and emphasize the frequency range of speech (Figure 2H) (Muda et al., 2010). Another model, directly relevant to physiology, is the Cochleagram (Brown and Cooke, 1994; Feather et al., 2019; Rahman et al., 2020). Cochleagrams mimic the cochlea by using a filter bank associated with points on the basilar membrane to mimic an impulse response Figure 2I).

A new approach that has shown much promise in bio-acoustics is Per-Channel Energy Normalization (PCEN; Figure 2J; Wang et al., 2017; Lostanlen et al., 2018). Lostanlen et al. (2018) identify three advantages of PCEN: (1) temporal integration, (2) adaptive gain control, and (3) dynamic gain compression. Temporal integration estimates the background noise at each frequency band. Adaptive gain control then adapts the gain of the spectral representation. Finally, dynamic range compression adaptively shifts the range of low and high amplitude components of the signal. Adaptive gain control is ubiquitous to mammalian auditory processing and is also often used in cochleagrams (Rahman et al., 2020). PCEN has been shown to aid in enhancing animal vocalizations relative to background noise across distances from the microphone (Lostanlen et al., 2019a) and reduce biases in bio-acoustics background settings such as dawn vs. dusk (Lostanlen et al., 2019b).

Descriptive basis-features features can also be used to represent vocalizations for downstream analyses. One challenge with using basis-features for vocal analysis is in determining what basis-features are relevant (Tchernichovski et al., 2000; Elie and Theunissen, 2016). Very few species have been rigorously examined to determine what acoustic features distinguish vocal units (Elie and Theunissen, 2016; Kershenbaum et al., 2016). Swamp sparrow notes, for example, are relatively simple vocalizations and can be well-described using just the length of the note, the peak frequency at the start of the note, and the peak frequency at the end of the note (Clark et al., 1987). One approach to determining what features are relevant in a vocal signal is to train classifiers to predict behaviorally-relevant information, such as individual identity, age, or the activity the animal is engaged on a full set to basis features, and retain those features which are highly informative (Elie and Theunissen, 2016, 2018).

## 4. Identifying, Segmenting, and Labeling Vocalizations

Vocalization data can be recorded in a number of different settings, ranging from single individuals in well-controlled and acoustically isolated lab settings to multi-individual and multi-species recordings taken next to a busy highway. When vocalizations are produced by isolated, single individuals, segmenting out vocalizations can often be performed simply by thresholding the vocal envelope and assuming any detected noise events that match the statistics of the vocalizing animal (e.g., frequency and length of vocalization) are vocalizations (Tchernichovski et al., 2000). More complex environments and species with more complex vocal structure require more complex solutions (Priyadarshani et al., 2018).

Experimental paradigms in neuroethology differ from bio-acoustics in that environmental sounds can usually be controlled, but are still faced with the challenge of often being made in colony settings with multiple vocalizing individuals or individuals who make non-vocal sounds such as interaction with a living space. Regardless of context, recent advances in machine learning algorithms for passive monitoring of acoustic environments allow for real-time labeling of species and individuals in noisy environments.

**Figure 3:**
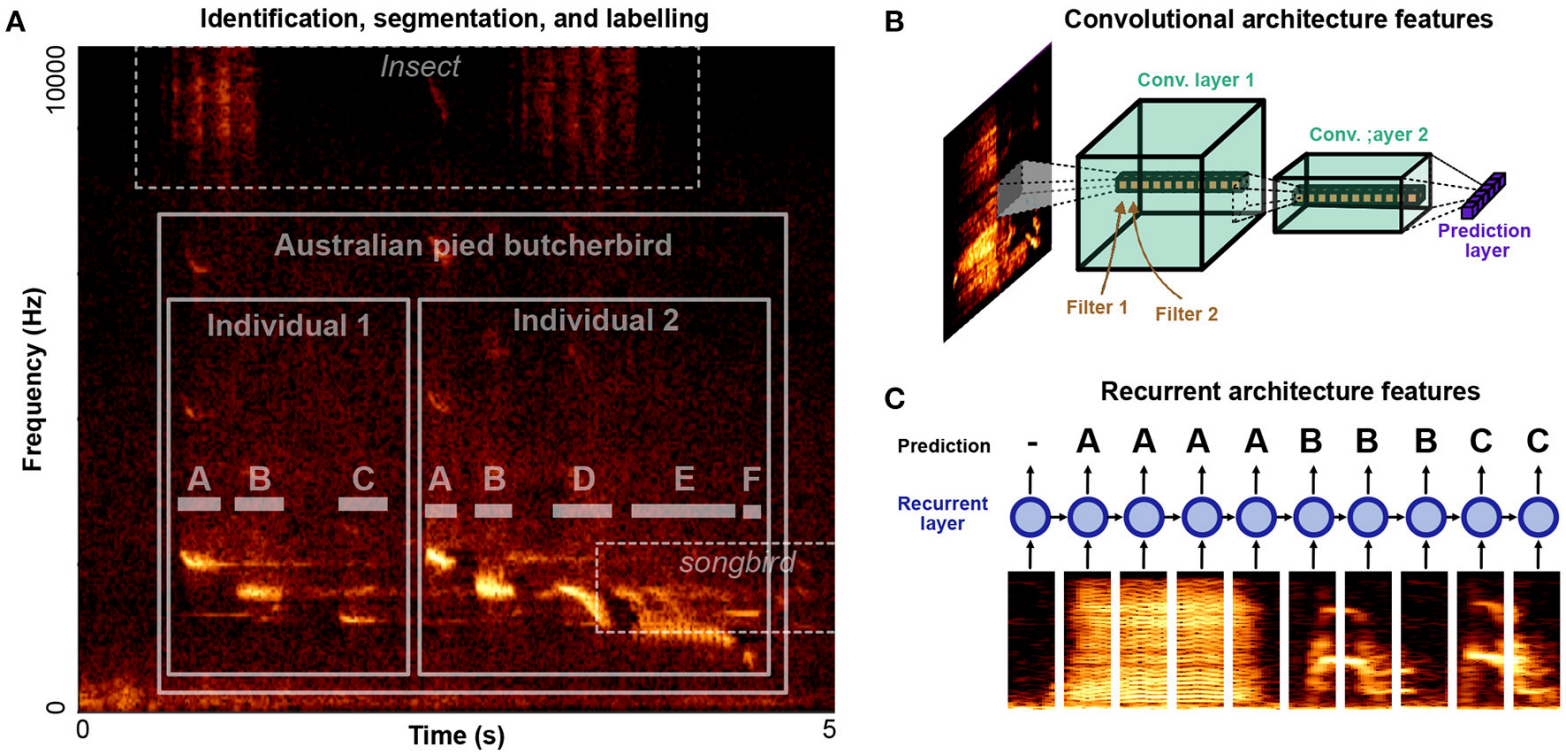
Levels of organization and architectural design features for identifying, segmenting, and labeling vocalizations. **(A)** An example clip of Australian pied butcherbird song (Janney et al., 2016) is shown containing two male butcherbirds, alongside background noise containing insect noise and another bird's song. A classification task exists at several levels: identifying the target species, differentiating individuals, detecting note boundaries, and classifying notes. **(B)** A cartoon diagram of a convolutional neural network architecture applied to a spectrogram. Convolutional filters are applied in time-frequency space. Deeper layers have larger spectrotemporal receptive fields and learn more complex filters. **(C)** A cartoon diagram of a recurrent neural network applied to a song spectrogram. Spectral slices are input to recurrent layers in the network (depicted as a circle) which are recurrent in time, allowing information to be integrated over time.

Automatic vocalization annotation can be broken down into three related tasks: identification, segmentation, labeling. Identifying refers to what animal is vocalizing and at what times and frequency channels. Segmentation refers to the segmentation of vocalizations into their constituent units, labeling then refers to grouping units into discrete element categories. A spectrogram outlining all three tasks is given in Figure 3A. Two individuals in the target species, Australian pied butcherbird *Cracticus nigrogularis* are vocalizing over top of background noise from another, unidentified, species of songbird, as well as an unidentified species of insect. Each bird's song can be divided into segmental units (notes) which can be further categorized into discrete element categories (“A,” “B,” “C,”...). In such a dataset, labeling challenges occur over multiple levels: identifying the species, identifying the individual, segmenting vocal units, and labeling vocal units into discrete categories. Some algorithms perform only one of these steps at a time, while others perform all three.

### 4.1. Detecting Species and Individuals

To detect species in continuous bio-acoustic data, several open-source tools and datasets have recently been made available for passive acoustic monitoring. A summary of many of these software and their features are given in Priyadarshani et al. (2018, Table 4). Over the past few years machine learning competitions challenging researchers to produce species recognition algorithms have motivated an increasing number of open-source approaches to bioacoustic sound recognition (e.g., Lasseck, 2013; Murcia and Paniagua, 2013; Goëau et al., 2014; Stowell et al., 2019). The same tools can be applied to differentiating between individuals in the same recording environment (e.g., Adi et al., 2010; Mielke and Zuberbühler, 2013). Most recent approaches rely on deep neural networks to detect vocalizations in noisy environments (e.g., Stowell et al., 2019; Cohen et al., 2020a). Current neural networks generally rely on some combination of convolutional filters in the temporal-frequency space of spectrograms (Convolutional Neural Networks or CNNs, Figure 3B) and temporal-recurrence (Recurrent Neural Networks, or RNNs, Figure 3C). Convolutional filters in the time-frequency space of spectrograms allow neural networks to learn complex spectro-temporal features used to classify sounds (Figure 3B). Temporal recurrence allows neural networks to learn sequential and temporal relationships that unfold over long time delays (Figure 3C). In combination, recurrent and convolutional architectures allow complex, non-linear spectrotemporal features that occur over arbitrary timescales to be captured by neural network architectures.

### 4.2. Segmenting and Labeling Vocal Units

Beyond identifying individuals and species, many analyses of vocal communication rely on the temporal segmentation and categorization of vocalizations into discrete units. Unlike identifying species or individuals, where an objective measure exists of what animal produced a vocalization, the segmental units that comprise animal vocalizations are less well-defined. In comparison to human language, where linguistic units are determined based on their functional role, substantially less is known about the function each vocal unit plays in most species' communication, or even what should define the beginning and ending of a vocal unit (Kershenbaum et al., 2016; Mizuhara and Okanoya, 2020). Analyses of most animals, therefore, rely on easily discernible physical features of vocalizations. For example in songbirds, songs are typically segmented at different hierarchical levels, though no strict definition of these levels of organization are agreed upon by all researchers. Common units of birdsong are notes, corresponding to abrupt changes in frequency, syllables, defined by periods of silence surrounding continuous vocalizations, motifs, stereotyped repetitive combinations of acoustic elements, and phrases, series of stereotyped or commonly associated syllables. Despite the ubiquity with which these terms are used, most vocal units have not been validated in terms of the species' own perceptual system, and those that do, like the Bengalese finch 'syllable' (Mizuhara and Okanoya, 2020) call into question the commonly assumed role they play in communication. It is therefore ideal, but not always feasible, to validate decisions about vocal units based upon perceptual, physiological, or functional roles those vocal units play in the animal's communication (Suzuki et al., 2006; Kershenbaum et al., 2016). Still, most analyses of animal communications rely on human perceptual decisions at some level, whether it is to label discrete classes of birdsong phrases, or determine the representational space upon which an “unsupervised” learning algorithm will discretize units (discussed in Section 5).

When vocal units are defined and vocal classes are chosen, machine learning algorithms can be used to systematize and vastly speed up the classification and segmentation of vocal units. Most commonly, supervised recognition algorithms are used, where the algorithm explicitly learns to algorithmically map acoustic data to the researcher's labeling scheme. Over the past decades, vocalization labeling algorithms have paralleled those used in other acoustic domains, such as speech and music recognition. At present, tools rely on deep neural networks. The field of deep learning has changed rapidly over the past decade, with different architectures of neural networks quickly outperforming the previous architectures (Nassif et al., 2019). Prior to deep learning, automated birdsong element recognition relied on algorithms such as Hidden Markov Models (Kogan and Margoliash, 1998), support vector machines (Tachibana et al., 2014), template matching (Anderson et al., 1996), or k-Nearest-Neighbors labeling (Nicholson, 2016), following alongside contemporary speech recognition algorithms. Like sound event detection, current approaches tend to rely on recurrent and convolutional neural network architectures. TweetyNet (Cohen et al., 2020a), for example, uses a recurrent and convolutional architecture to capture complex spectro-temporal patterns over long timescales. Future advances in neural network architectures will likely continue to follow those in speech recognition, for example, using transformer architectures (Karita et al., 2019) as well as semi-supervised and unsupervised pre-training methods such as wav2vec (Schneider et al., 2019). One important divergence between speech recognition and animal vocalization classification is the reliance upon data availability, however. An ideal animal vocalization classifier works well on very small amounts of labeled data, requiring less experimenter time, whereas speech recognition systems tend to have an abundance of data available (though speech recognition for low-resource languages may be an area to watch).

A second approach to labeling vocalizations is to actively involve the experimenter in the algorithm via human-in-the-loop labeling (e.g., Wimmer et al., 2010; Kim and Pardo, 2018). Human-in-the-loop algorithms rely on a combination of supervised and unsupervised learning. Supervised learning comprises learning algorithms that are trained with labeled data, such as classification tasks. Unsupervised learning refers to algorithms that do not require supervised labels, such as dimensionality reduction. Human-in-the-loop algorithms leverage both, by proposing an initial coarse segmentation and/or labeling of the dataset through unsupervised learning, which the human then partially revises (e.g., merging or splitting putative classes of vocalizations) via a graphical user interface (GUI). The revised data is then re-processed by the algorithm and sent back to the user to revise, until the experimenter is content with the resulting labeled dataset. Using a combination of human expertise and machine processing enables quicker labeling of large bio-acoustics data with minimal human effort. A further discussion of unsupervised algorithms is discussed below in Section 5.

## 5. Extracting Relational Structure and Clustering

Classifying vocal elements into discrete categories (e.g., “A,” “B,” “C,”...) is for many analyses a necessary abstraction that enables the analysis of recurring events. At the same time, this symbolic abstraction ignores acoustic relationships both within discrete element categories and between them. For example, in Figure 3, are the syllables of birdsong Figure 3A more similar to the syllables Figure 3B or the syllables Figure 3C? Determining the relatedness (or distance) between vocalizations can enable the quantification of how vocalizations change over time (Mets and Brainard, 2018; Kollmorgen et al., 2020), how vocal repertoires differ across individuals and species (Miller, 1979; Sainburg et al., 2020b), and map and visualize broad structure present in vocal repertoires (Sainburg et al., 2020b; Goffinet et al., 2021).

### 5.1. Operationalizing Relatedness

Given a dataset of vocalizations segmented into discrete units, relatedness is a measure quantifying the similarity of vocalizations relative to one another. The basis for operationalizing relatedness can utilize physical properties of signals, perceptual judgments, or behavioral and physiological responses to the signal. Most commonly, the relationships between vocal elements are computed on either spectrotemporal representations or on the basis of descriptive features of the vocalization, such as frequency modulation, fundamental frequency, and vocal envelope (Miller, 1979; Sainburg et al., 2020b; Goffinet et al., 2021).

How different aspects of the vocalization should weigh into a measure of similarity is non-trivial. No metric for similarity is objectively correct, even when metrics are derived purely from objective physical features. For example, what is the relative importance of a vocalization's duration vs. fundamental frequency in determining similarity? One ground truth metric for an algorithms judgement of similarity is its relationship with human's perceptual judgment of similarity (Tchernichovski et al., 2000), though there is no guarantee that these measures reflect the animal's own perception and physiology (Dooling and Prior, 2017). An ideal measure of similarity could be derived through careful experimentation gleaning the animal's own judgment of similarity (Kershenbaum et al., 2016), but in most cases, this task would be unfeasible and time-consuming. Even when performed carefully, perception varies from animal to animal, based upon experience (Lachlan et al., 2010).

In addition, when vocal features are continuous, accounting for differences in duration and temporal alignment requires consideration. Approaches vary from averaging over time (Elie and Theunissen, 2016), pooling using attention mechanisms (Morfi et al., 2021), using dynamic time warping (Kogan and Margoliash, 1998), and zero-padding (Sainburg et al., 2020b). Similarly, at least some animals rely on spectral shape rather than absolute pitch when recognizing acoustic objects (Bregman et al., 2016). A recent approach accounting for variability in frequency is dynamic frequency warping (Somervuo, 2019). Striking a balance between spectrotemporal tolerance and absolutely discounting spectrotemporal alignment can have substantial impact on the final measure of similarity.

### 5.2. Learning a Similarity Space

Once a metric for similarity is determined, that distance can be used to infer a structured representation of the relationships in a repertoire as a whole, providing a new representational space with which to quantify vocalizations.

Perhaps the most intuitive and pervading example of a learned embedding space for vocal similarity is multi-dimensional scaling [MDS, e.g., (Miller, 1979; Dooling et al., 1987; Morfi et al., 2021)]. Multi-dimensional scaling takes a graph of pairwise similarity measures between each vocalization in the dataset and attempts to find an embedding that best preserves the similarity structure of that graph. As the number of vocal elements in a dataset gets larger, however, the number of pairwise distances between vocal elements increases exponentially. This is computationally an issue because computing 10,000 pairwise distances between 100 elements is computationally feasible, but 10,000,000,000 pairwise distances between 100,000 elements is not.

**Figure 4:**
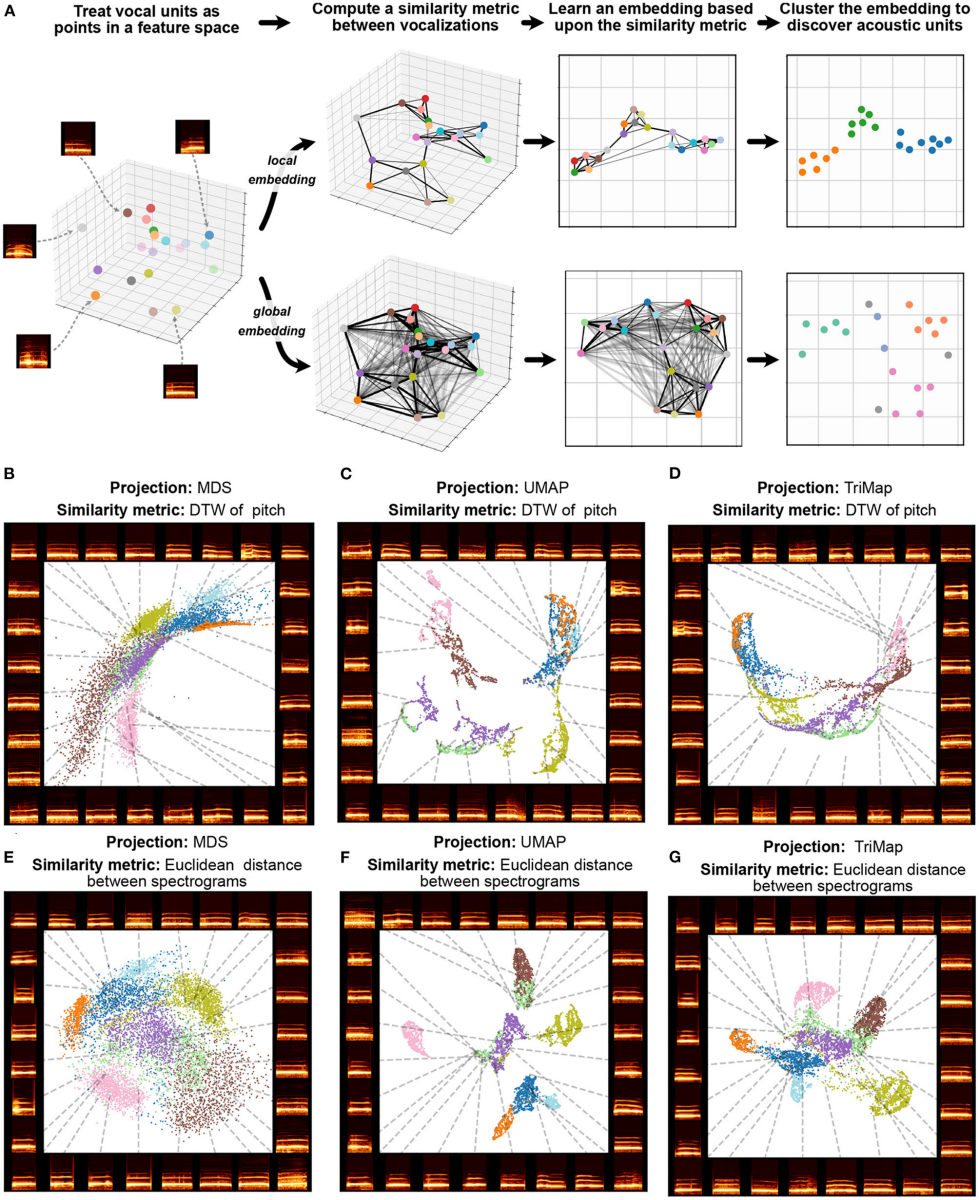
Local and global embeddings. **(A)** The steps outlined in Section 5 exhibit the differences between the relationships preserved in local and global embeddings. **(B–D)** Projections of a dataset of macaque coo calls (Fukushima et al., 2015) using two similarity metrics (Dynamic Time Warping over frequency, and Euclidean distance between spectrograms) and three projection algorithms (Multidimensional Scaling, UMAP, and TriMap) **(E–G)**. Colors represent individual identity.

Trying to preserve the pairwise relationships between every element in a dataset can also over-emphasize irrelevant relationships in vocal data. For example, if a bird's vocal repertoire comprises 10 motifs classes all produced with the same frequency, the vast majority of pairwise distance relationships computed (90%) will be between class, while only 10% of pairwise relationships computed will be within class. In many cases, both in animal communication and in dimensionality reduction more broadly, there is utility in weighing relationships between similar vocal elements more highly than relationships between less similar vocalizations. This contrast is defined in the dimensionality reduction literature as the emphasis of local vs. global structure (De Silva and Tenenbaum, 2002). Algorithms that attempt to preserve every pairwise relationship are called global dimensionality reduction algorithms, while algorithms that emphasize capturing relationships only to nearby points in dataspace (more similar vocalizations) are called local dimensionality reduction algorithms. In many vocalization datasets, emphasizing local over global structure better preserves categorical structure such as individual and call identity (Sainburg et al., 2020c; Goffinet et al., 2021; Morfi et al., 2021). A visual demonstration contrasting local and global structure preservation is given in Figure 4A. While global embedding algorithms like MDS attempt to preserve every pair-wise relationship, local algorithms preserve only local (e.g., nearest-neighbor) relationships, capturing more within-cluster structure. In Figures 4B–G an example is given with macaque coo calls, in which a local structure-preserving algorithm (UMAP, described below) more clearly pulls apart clusters corresponding to individual identity than MDS.

At present, the two dominant local dimensionality reduction algorithms are UMAP and t-SNE. UMAP and t-SNE differ in several important ways beyond the scope of this paper, but their key intuition and the steps underlying the algorithms remain similar: first, compute a (nearest-neighbors) graph of pairwise relationships between nearest neighbors in the original dataset (e.g., using Euclidean distance or an arbitrary similarity metric) then, embed that graph into an embedding space via gradient descent (Sainburg et al., 2021). UMAP, in particular, has been shown to capture complex structure in vocal repertoires such as differences in vocal dialect, vocal stereotypy, vocal element categories, inter-species similarity, and individual identity, in contrast to classic methods like MDS and PCA (Goffinet et al., 2021; Morfi et al., 2021; Sainburg et al., 2021).

One challenge with graph-based dimensionality reduction algorithms like MDS, UMAP and t-SNE is that they are non-parametric dimensionality reduction algorithms, meaning they do not learn the relationship between input data (e.g., a spectrogram of the vocalization) and their embeddings. Learning a parametric relationship between vocalizations and their embeddings allows a fast mapping between data and embedding, i.e., for applications that necessitate real-time feedback such as brain-machine interfacing.

The most common parametric dimensionality reduction algorithm is PCA, where a linear transform is learned between data and an embedding space. Similarly, neural networks such as autoencoders can be used to learn a set of basis features which can be complex and non-linear (Kohlsdorf et al., 2020; Sainburg et al., 2020c; Goffinet et al., 2021; Singh Alvarado et al., 2021). For example, an autoencoder trained on images of faces can learn to linearize the presence of glasses or a beard (Radford et al., 2015; Sainburg et al., 2018b, 2021). Autoencoders trained on animal vocalization data can similarly learn complex non-linear relationships in vocal data. In Section 8 we discuss how these complex learned features could be utilized in animal vocalizations to learn acoustic features such animal age, sex, and attractiveness, which can, in principle, be utilized for playback experiments.

A recent extension to UMAP, Parametric UMAP weds the advantages of UMAP with the parametric embedding of neural networks (Sainburg et al., 2021). Parametric UMAP acts by optimizing the UMAP loss function over arbitrary neural networks (e.g., convolutional recurrent networks were used with Cassin's vireo song in Sainburg et al., 2021) which can be balanced with additional losses such as MDS and autoencoding, to preserve additional global structure in UMAP projections. Parametric neural network-based approaches such as Parametric UMAP can also embed data on a similar timescale as PCA, enabling real-time applications, as opposed to non-parametric methods such as UMAP, t-SNE, and MDS.

Another class of neural network based dimensionality reduction algorithms rely on triplet-loss-based similarity preservation. Triplet-based embeddings have been used for birdsong for classification and embedding (Morfi et al., 2021; Renteria et al., 2021). Triplet networks learn an embedding space by sampling three types of vocal units: an anchor, a positive sample that is perceptually similar to the anchor point, and a negative sample that is perceptually distant from a vocal unit. The loss then encourages the positive sample to be pulled to the anchor, and the negative sample to be pushed further away. For example, Morfi et al. (2021) describe a triplet-loss-based network trained to produce vocal embeddings based upon a metric of perceptual distances. Like graph-based dimensionality reduction algorithms, triplet-loss-based embeddings rely on a pre-defined experimenter-determined notion of distance. Morfi et al. suggest a forthcoming animal-defined metric but in-lieu use a descriptive feature-based metric in the software Luscinia (Lachlan, 2007) which is correlated with human perceptual judgments of zebra finch song (Holveck et al., 2008).

### 5.3. Finding Latent Units Through Clustering

Learned embedding spaces enable the inference of broad structure acoustic structure from the statistics of vocalizations, enabling further downstream discovery of vocal units based upon distributional properties in embedding spaces (Kershenbaum et al., 2016; Sainburg et al., 2020b; Keen et al., 2021). Unsupervised clustering of vocal elements lies in contrast with supervised learning, where class labels are determined by experimenters, as in Section 4. Sainburg et al. (2020b) observe that labels obtained by clustering UMAP embeddings of Cassin's vireo and Bengalese finch syllables are more similar to experimenter labels than clustering PCA projections or spectrograms. Further, these latent projections capture additional acoustic and syntactic structure than the ground truth experimenter labels. In addition to acoustic structure, vocal elements can be clustered on the basis of syntactic organization. For example, incorporating transition information through Partially observable Markov Models (POMMs; Jin and Kozhevnikov, 2011) and Hidden Markov Models (HMMs; Katahira et al., 2011; Sainburg et al., 2020b) into a labeling scheme for birdsong better explains sequential structure than hand-labels or clustering without reference to temporal sequencing. An alternative approach is to perform clustering prior to embedding, directly upon the inferred relational graph (Frasier et al., 2017).

One challenge in unsupervised vocal unit discovery through methods such as UMAP embeddings is their reliance upon pre-defined vocal unit temporal boundaries. Although clustering on latent projections enables an unsupervised extraction of vocal categories from segmental units, latent projections rely on a pre-defined temporal segmentation of acoustic units from the vocal stream. In some species, atomic vocal units can be determined by clearly defined physical features of the signal, like long pauses between syllables, however, even in the case of clearly defined physical features, those units are not necessarily the base units of perception (Mizuhara and Okanoya, 2020). An open issue in vocal analysis is the unsupervised temporal segmentation of vocalizations into elements when clear physical boundaries are not available. This problem parallels both unsupervised speech discovery (i.e., ZeroSpeech), and the challenge of discovering behavior units in other areas of computational neuroethology (e.g., Motion Sequencing). In speech, phonemes are not clearly defined by physical characteristics, thus approaches for segmentation rely upon a combination of temporal and distributional information alongside imposed priors. Ongoing efforts in unsupervised speech segmentation, syllabic unit discovery, and word discovery can motivate parallel approaches in animal communication. In addition, physiological and kinematic measures such as articulation and breathing rate can aid in determining vocal boundaries. In computational neuroethology, new methods in tracking behavioral kinematics provide similar continuous behavioral datasets to those discussed in this paper (e.g., Wiltschko et al., 2015, 2020; Berman et al., 2016; Mathis et al., 2018; Pereira et al., 2019; Dunn et al., 2021; Marshall et al., 2021). For example, MoSeq (Wiltschko et al., 2015, 2020) discovers animal behavioral states using depth camera recordings of animals by fitting the behavioral data to an Autoregressive Hiden Markov Model. They find stereotyped sub-second mouse behavioral states, dubbed syllables, that underlie a syntax of behavior, much like birdsong. Communicative behavior is also not produced solely in the auditory domain. Improving methods for uncovering structure in animal behavior more broadly will facilitate research on the interaction between multi-sensory and multi-modal vocal behavior, like the dances that accompany many bird songs (Williams, 2001).

### 5.4. Data Augmentation

Another approach that is largely underutilized in bio-acoustic vocal recognition algorithms is data augmentation, an approach that is currently used in most state-of-the-art machine perception applications. In automatic speech recognition, for example, several current state-of-the-art approaches (e.g., Baevski et al., 2020; Gulati et al., 2020) use SpecAugment (Park et al., 2019) in which the classifier learns a policy of various augmentations such as warping and masking frequency channels in time. Lostanlen et al. (2019b) demonstrate the utility of augmenting bio-acoustics datasets with diverse background acoustics to facilitate better generalization across environments and conditions. Augmentation in settings where little labeled data are available has also proven successful on several semi-supervised learning benchmarks (e.g., Berthelot et al., 2019). One difficulty with performing data augmentation with bio-acoustics data, however, is the extent to which slight manipulations can affect the perceptual class that vocalizations fall into (Morfi et al., 2021).

## 6. Inferring Temporal and Sequential Structure

Identifying sequential organization typically relies upon the abstraction of vocalizations into discrete sequences of elements, effectively treating vocal data as corpora from which to perform symbolic analyses. Kershenbaum et al. (2016) identify six classes of models and analyses for analyzing temporal sequences: Markov chains, Hidden Markov Models, Network-based analyses, Formal grammars analyses, and temporal models. Analyses of temporal organization in animal communication has traditionally been largely influenced by Chomsky's hierarchy of formal grammars, with a focus on trying to understand what class of the Chomsky hierarchy animal's behaviors belong within (Hauser et al., 2002; Rohrmeier et al., 2015; Jiang et al., 2018; Morita and Koda, 2019). For example, Markov models, Hidden Markov Models, and Network models are all finite-state models in the Chomsky hierarchy.

### 6.1. Short-Timescale Organization and Graphical Analysis

**Figure 5:**
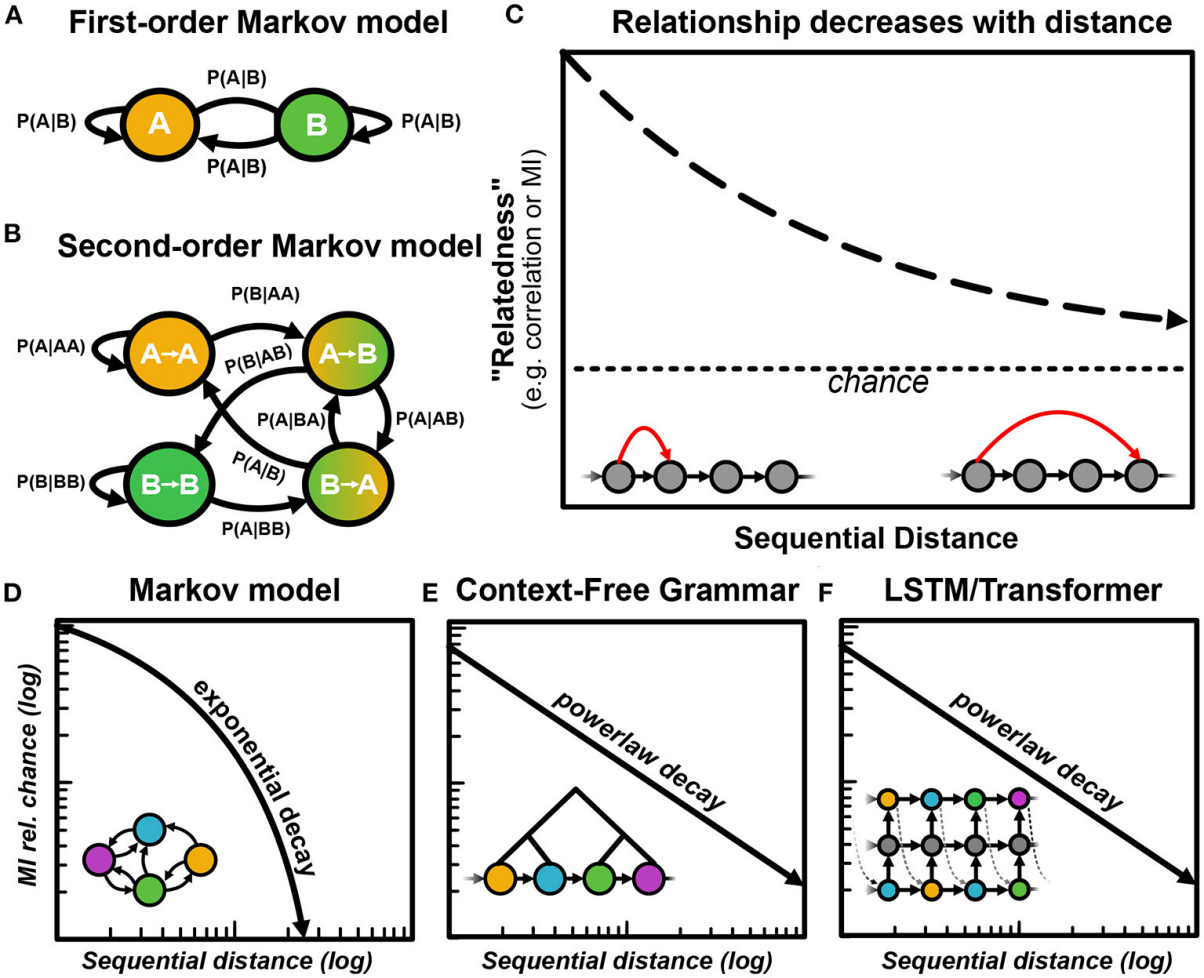
Capturing long and short-range sequential organization with different models. **(A)** An example of a 2-state Markov model, capturing 2^2^ = 4 transitional probabilities between states. **(B)** An example second-order Markov model, capturing 2^3^ = 8 transition probabilities between states. **(C)** A visualization of the general principle that as sequential distances increase, the relatedness between elements (measured through mutual information or correlation functions) decays toward chance. **(D)** Sequences generated by Markov models decay exponentially toward chance. **(E)** Context-free grammars produce sequences that decay following a power law. **(F)** Certain neural network models such as LSTM RNNs and Transformer models produce sequences that also decay following a power law.

Broadly, analyses over vocal organization can be broken down into two classes: analyses over short- and long-distance (i.e., short- and long-timescale) sequential organization. Short-timescale analyses are concerned with relationships between adjacent, or near adjacent elements in a sequence. Markov models, for example, capture short-timescale dynamics of vocal communication. A typical Markov model of birdsong is simply a transition matrix representing the probability of transitions from each element to each other elements [e.g., *P*(*B*|*A*) Figure 5A]. As Markov models increase in order, they become increasingly capable of capturing long-distance relationships, though high-order Markov models are rarely used in practice because of the number of parameters and amount of data needed to compute them (Figure 5B). Approaches such as Hidden Markov Models (Katahira et al., 2011) and Probabilistic Suffix Trees (Markowitz et al., 2013; Cohen et al., 2020b) can compute more succinct high-order Markov relationships, though the amount of data needed to capture these deeply contextualized relationships (e.g., *P*(*F*|*A, B, C, D*)) is still a limiting factor in capturing long-range organization with Markov models. Short-range relationships are also often captured graphically, treating any transition probability above zero as an edge in the graph. Graphical representations and metrics for vocal sequencing can explain general sequencing characteristics of vocalizations such as network motifs, communities, and clusters (Sasahara et al., 2012; Weiss et al., 2014; Hedley, 2016; Kershenbaum et al., 2016; Patricelli and Hebets, 2016).

### 6.2. Mutual Information and Long-Timescale Organization

Relationships that extend beyond adjacencies and over longer timescales are called long-range orlong-timescale relationships. For example, how related are two notes within a phrase, two phrases within a bout of song, or two bouts of song sung within a day?

Broadly, elements that are further displaced in a vocalization from one another tend to be less related. When two elements in a sequence are further apart, the relatedness between those two elements tends to be lower. For example, in birdsong, notes within a phrase are more likely to be related than notes separated by multiple phrases. The same is true of most sequential and temporal data: we can better predict what a stock price will look like tomorrow, than in 10 years. As we look further and further out into a sequence, the relatedness between elements will decrease alongside our ability to predict the future, until the relatedness approaches chance (Figure 5C). We can capture this relatedness over symbolic sequences using information theory. For example, given a sequence of discrete elements *a* → *b* → *c* → *d* → *e* → *f*, we can estimate the mutual information between pairs of elements at e.g., a distance of 2 elements (*a* − *c*, *b* − *d*, *c* − *e*, and *d* − *f*) or 3 elements (*a* − *d*, *b* − *e*, and *c* − *f*). As the distance increases between pairs of elements, we expect the relatedness (mutual information) to decay toward chance as a function of sequential distance.

We can estimate the extent to which a signal exhibits long-range relationships by computing how long the mutual information between pairs of elements remains above chance. Such approaches have been used variously across animal vocalization datasets in birds and whales (Suzuki et al., 2006; Sainburg et al., 2019). Similar approaches have also been used to observe long-range structure in animal motion ethology data, such as the long-range structure in *Drosophila* (Berman et al., 2016) motility.

### 6.3. Inferring Structure From Sequential Relationships

The shape of the decay in relatedness as a function of sequential distance can not only tell us about the timescales that vocal sequences are operating over but can also give indications about the structure underlying sequential organization. For example, sequences generated by Markov processes, such as finite-state grammars decay exponentially (Li, 1990; Lin and Tegmark, 2017) (Figure 5D). Intuitively, Markov models are memoryless; each state is dictated only by the set of transition probabilities associated with the previous state. As a result, the relatedness between states decays very quickly. When there are deep latent relationships present in the structure underlying the sequence, relatedness between sequentially disparate elements decays more slowly. For example, Probabilistic Context-Free Grammars can produce power-law relationships in mutual information as a function of sequential distance (Lin and Tegmark, 2017) (Figure 5E).

Characterizations of statistical relationships over abstracted discrete units enables comparative analyses across species because these measures make no assumptions about units or temporal organization underlying the signal. Characterizing correlations and information decay has an especially rich history in uncovering long-range structure dating back to Claude Shannon's original work (Shannon, 1951; Li, 1990; Lin and Tegmark, 2017). Language corpora such as speech and written text decay in information following the combination of a power-law over longer distances, and exponential decay over shorter distances, attributed to the finite-state processes underlying phonological organization (Sainburg et al., 2019) and the hierarchical organization underlying language at higher levels of organization such as syntax and discourse (Alvarez-Lacalle et al., 2006; Altmann et al., 2012; Lin and Tegmark, 2017; Sainburg et al., 2019). At the same time, however, young children's speech contains the same long-range information context before complex syntax is present in speech, indicating possible extra-linguistic mechanisms at play dictating these long-range statistical relationships (Sainburg et al., 2020a). Long-range mutual information decay and correlations have also been demonstrated that in animals such as songbirds (Sainburg et al., 2019) and humpback whales (Suzuki et al., 2006), extending over minute- and hour-long timescales. In particular, birdsong exhibits similar exponential short-range and power-law long-range mutual information decay to human speech, indicating potential parallels in the mechanisms governing how patterns of vocalizations are temporally sequenced. Similar observations in non-vocal behavioral sequences (Berman et al., 2016; Sainburg et al., 2020a) also exhibit these long-range sequential organizations, suggesting similarities in latent dynamics that facilitate long-range statistical relationships.

It is tempting to suggest that these parallels suggest shared underlying structure generating mechanisms, such as universals in the hierarchical organization of behavior (e.g., Lashley, 1951; Dawkins, 1976), though we should be wary of making any extended inferences based upon the observation of long-range information decay. For example, we can infer that power-law sequential relationships are produced by non-Markovian mechanisms because the decay is not exponential. However, the set of generative mechanisms that can produce power-law relationships in signals is not understood well enough to attribute the origins of these relationships to, for example, any specific class of formal grammar. Power-law mutual information decay in signals can also be drawn simply from coupling vocal or behavioral 1/f noise found in exogenous environmental signals.

While it is well-acknowledged that many animal vocalizations are organized hierarchically (Dawkins, 1976; Rohrmeier et al., 2015), the implications of that hierarchy in terms of underlying cognitive and physiological mechanisms are not well understood. For example, on very short timescales, birdsong motor sequencing is dictated by a hierarchical cascade of motor programs running originating in the premotor region HVC eventually ending in motor output (Doupe and Kuhl, 1999). Recent physiological evidence shows that these high-level nuclei also contain information about future states displaced from current vocalizations as well (Cohen et al., 2020b), though the mechanisms by which those relationships are learned, maintained, and ultimately dictate behavior are not yet clear.

Although we do not have access to the mechanisms underlying the observed long-distance relationships in vocal and non-vocal behavioral sequences, we do know that many vocal and behavioral sequences cannot be well-captured by Markovian models, thus alternative methods for modeling, characterizing, and forming hypotheses about the long-range organization in behavioral sequences are crucial to furthering our understanding of long-range structure in behavioral sequences. One promising approach is the use of deep neural network models such as RNNs and transformer networks (Tran et al., 2018; Morita et al., 2020). Unlike Markov models, recent neural network models like RNNs and transformer models do capture power-law relationships in sequential data (Figure 5F) (Lin and Tegmark, 2017; Shen, 2019). In language, transformer networks, in particular, have changed the landscape of natural language processing by capturing deeply contextual and complex implicit relationships in linguistic sequences. In birdsong, the same approaches show promise (Morita et al., 2020). For example, Morita et al. (2020) train a transformer network on Bengalese finch song and find that it captures long-range dependencies extending well beyond that of a Markov model. Like modeling language sequences, however, neural-network-based approaches suffer from the same issues of being black box and providing little explanatory power over the sequential structure they learn. In addition, the amount of data required to train a model to capture complex sequential dependencies is vast. Although the number of parameters does not increase exponentially with the amount of context the model captures (as in Markov models) state-of-the-art transformer models have billions of parameters requiring training data comprised of billions to trillions of characters (Brown et al., 2020). In language, the dataset size needed to train transformer models scales with the number of parameters in the model to prevent overfitting (Kaplan et al., 2020). When dataset sizes are smaller, LSTM RNNs perform better than more state-of-the-art Transformer language models (Ezen-Can, 2020), though transformers allow you to explicitly specify the length of temporal context allowed in the model, making them an attractive model for controlling context when generating vocal sequences (Morita et al., 2020). Although non-human animal vocalization repertoires are smaller and syntactic organization is less complex than language, birdsong analyses relying on language models will need to address the same challenges.

Neural network-based models also provide the ability to capture temporal dependencies that mutual information and correlation functions do not. Mutual-information-function-based and correlation-based analyses compute relationships between vocal elements as a function of sequential distance, ignoring any temporal relationships between disparate elements. This is both a benefit and a shortcoming of correlation methods. Ignoring intermediary temporal relationships enables the characterization of structure at temporal distances without having to additionally model higher-order combinatorial relationships [e.g., *P*(*F*|*A*)) vs. *P*(*F*|*A, B, C, D*)]. For the same reason, mutual-information-function-based and correlation-based analyses are coarse descriptions of temporal structure and miss the full temporal dynamics of the signal that neural-network-based models can capture (Morita et al., 2020).

## 7. Synthesizing Vocalizations

Although the methods discussed in Section 5 allow us to learn representational spaces of animal vocalizations, providing new ways to infer structure in vocal repertoires, analyses on vocal signals alone lack grounding in animal behavior, perception, and physiology. In this section, we give an overview of methods for synthesizing animal vocalizations as a means to systematically control vocalization stimuli and relate vocal representations to physiology and behavior.

An ideal model for vocal synthesis exhibits several features: (1) it can model the entire vocal repertoire of a species or multiple species, (2) the parameters of the model can be related to physiological properties of the vocalizing species, and (3) the parameters of the model can be explained in terms of understandable features (i.e., it is not a black box algorithm). Throughout this section, we find that current synthesis algorithms have tradeoffs in how they balance aspects of these ideals.

One reason to systematically synthesize animal vocalizations is to probe their perceptual and physiological representations of vocal space, for example, determining how animals categorically perceive the difference between two categories of vocal units (Nelson and Marler, 1989). Traditionally, categorical perception in animals has been studied on the basis of human speech sound stimuli (Sinnott et al., 1976; Kuhl and Miller, 1978; Kuhl and Padden, 1983). Even with speech, however, the features that can be manipulated are limited. Recently methods in machine learning have furthered our ability to manipulate complex non-linear speech features substantially. These same approaches can often be applied to animal communication (Anikin, 2019; Sainburg et al., 2020c).

### 7.1. Source-Filter Models

Source-filter models have their origins in vocoding speech (Dudley, 1939), but have been used in numerous animal vocalization synthesis paradigms (DiMattina and Wang, 2006; Chakladar et al., 2008; Arneodo and Mindlin, 2009; Furuyama et al., 2017). Source-filter models decompose vocalizations into the source of the voice and filters (Kawahara, 2006). For example, the STRAIGHT algorithm (Kawahara et al., 1999; Kawahara, 2006) has been used to morph between macaque monkey vocalizations for investigations of monkey and human perception and physiology related to categorization (Chakladar et al., 2008; Furuyama et al., 2017). STRAIGHT breaks down the macaque vocalization into the fundamental frequency (the source) and its harmonics from higher-resonant or formant frequencies (the filter) (Chakladar et al., 2008). It then uses landmarks based upon these estimated parameters from the two sounds being morphed and interpolates between them to generate the morph stimuli. Furuyama et al. (2017), for example, used this method to parametrically vary generated morphs based on source and filter properties to determine the features macaques use to distinguish between conspecifics. Soundgen (Anikin, 2019) is a recent open-source GUI-based web app for R that is designed to synthesize nonverbal vocalizations using a source-filter model, including animal vocal signals such as birdsong and primate vocalizations. Related source-filter models have been developed to synthesize birdsong based upon underlying physiological mechanisms (Fee et al., 1998; Sitt et al., 2008, 2010; Arneodo and Mindlin, 2009; Arneodo et al., 2012). Recently, Arneodo et al. (2021) demonstrated that synthetic source-filter models can be coupled with neural recordings accurately reconstruct vocalizations from neural data alone. One drawback of source-filter models is the difficulty with which they can be fitted to the diversity of non-human vocalizations that exist. For example, the source-filter models of birdsong described above can well describe the dynamics of zebra finch song, but not the dual-syringeal dynamics of European starling song. Without reference to explicit hypotheses about underlying production mechanism, HMM based source-filter approaches provide one potential solution to this problem birdsong (Bonada et al., 2016).

### 7.2. Neural Network Models

**Figure 6:**
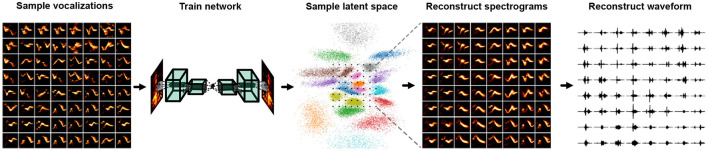
Steps involved in synthesizing vocalization from a Variational Autoencoder (VAE) trained on spectrograms.

An alternative approach to synthesizing animal vocalizations is the use of neural-network-based synthesis algorithms. These neural-network-based algorithms can be used to sample directly from the learned representational spaces described in Section 3. A simple example is autoencoder-based synthesis (Figure 6) (Sainburg et al., 2018a; Zuidema et al., 2020). Autoencoders can be trained on spectral representations of vocal data, and systematically sampled in the learned latent space to produce new vocalizations. Insofar as the neural network or latent projection can learn to represent the entire vocal repertoire, the entire vocal repertoire can be sampled from. In addition to sampling vocalizations from a latent distribution, vocal features can be manipulated in latent space. Well-defined latent spaces and higher-dimensional latent projections can learn to linearize complex non-linear reltionships in data. For example, in pictures of faces, the presence of a glasses, hair color, and the shape of a person's face can all be manipulated as linear features (Radford et al., 2015; Sainburg et al., 2018b, 2021). With more complex features, such as the attractiveness of a call or the age of the vocalizer, a promising avenue for future research would be to synthesize vocalizations, varying these complex non-linear features for playback studies.

Like most areas of deep learning, substantial progress has been made on the task of audio synthesis in the past few years. Basic methods comprise autoencoders (Engel et al., 2017; Kohlsdorf et al., 2020; Sainburg et al., 2020c), Generative Adversarial Networks (GANd) (Donahue et al., 2018; Engel et al., 2019; Sainburg et al., 2020c; Tjandra et al., 2020; Pagliarini et al., 2021) and autoregressive approaches (Mehri et al., 2016; Oord et al., 2016; Kalchbrenner et al., 2018; Prenger et al., 2019). One advantage of GAN-based models is that their loss is not defined directly by reconstruction loss, resulting in higher-fidelity syntheses (Larsen et al., 2016). Typically, approaches for synthesizing vocalizations based on neural networks rely on treating magnitude spectrogram like an image, training a neural network architecture in the same manner as one would an image, and finally inverting the sampled spectrogram into a waveform (Sainburg et al., 2020b; Zuidema et al., 2020; Pagliarini et al., 2021). When synthesizing vocalizations from neural networks trained on the magnitude spectrogram, the estimation of phase is necessary to invert the spectrogram into a waveform signal for playback. The de-facto algorithm for spectral inversion has been Griffin and Lim (Griffin and Lim, 1984), though several recent approaches have been shown to improve over the Griffen-Lim algorithm recently (Prša and Rajmic, 2017; Masuyama et al., 2019). An alternative to Griffen-Lim inversion is to train neural networks to invert spectrograms either directly in the neural network architecture (Kumar et al., 2019), or perform inversion in a second network (Masuyama et al., 2019). Spectrogram-based audio synthesis can also be sidestepped entirely, training the network directly on waveform (Mehri et al., 2016; Oord et al., 2016; Engel et al., 2017).

### 7.3. Sound Texture Synthesis

Another approach to sound synthesis is the synthesis of sound texture (Saint-Arnaud and Popat, 1995; McDermott et al., 2009). For example, McDermott et al., (McDermott et al., 2009) propose an approach that relies on computing a set of statistics over stationary elements of sounds, and synthesizing new sounds based upon the computed statistics. By manipulating or interpolating between sound statistics, they synthesize new sound textures. One application, for example, is to manipulate components of sound textures for stimulus playback to determine what sound texture statistics listeners rely upon for recognition (McDermott and Simoncelli, 2011).

### 7.4. Generating Sequences

A parallel approach to synthesizing vocalizations is to generate vocal sequences from symbolically labeled vocal elements. Synthetic song sequences can be used to understand how animals process and represent temporal and sequential organization. For example, can a songbird differentiate between sounds generated using different underlying models of song syntax? Traditional approaches to song sequence generation rely upon the explicit, hand-crafted, generation of artificial grammars for playback studies. By crafting artificial grammars that differ in underlying structure, such as belonging to different classes of the Chomsky hierarchy (Gentner et al., 2006; Fitch and Friederici, 2012; Kershenbaum et al., 2014), playback studies can be used to determine whether animals can learn these grammars. A number of challenges exist with artificial grammar learning studies, however. One such challenge is the difficulty in crafting sequences that can exclusively be learned by inferring the structure that generated them, for example, making it impossible for the animal to learn by brute-force memorizing every sequence (Fitch and Friederici, 2012). When using artificial grammars, computational and modeling considerations aid in forming hypotheses about how generated grammars can be used. In the context of the neuroethology of vocal communication, these cognitive models can be related to physiological measures (Zuidema et al., 2020). An additional challenge with artificial grammar learning is constructing sequences that are structured in a similar way to natural and behaviorally relevant signals to the animal. For example, artificial grammar studies usually rely on short sequences modeled after human language syntax, rather than the animal's own communication systems. Because the task of generating vocal sequences is performed over symbolic representations of syllables, generating vocal sequences can be performed using the same methods as in text or musical note generation. These approaches can range from generating sequences using Markov models of various orders, to explicitly modeling hierarchical organization in the signal generation algorithm (Roberts et al., 2018).

## 8. Mapping Vocal Communication to Perception, Behavior, and Physiology

The methods discussed here provide a framework to develop a set of constrained spaces from which to understand and model vocal behavior in relation to perception, production, and physiology. Perceptual or relational vocal spaces, such as UMAP projections of spectrograms, provide a low-dimensional space that can be used to infer structure in vocal repertoires. Likewise, symbolic abstractions of vocal behavior to large corpora provides a categorical representation in which vocal behavior is seen as sequential actions on those category sets. In both cases, the methods provide a constrained behavioral representation for physiological analyses.

### 8.1. Brain-Computer Interfacing

One of the primary challenges facing the field of brain-computer interfacing is scaling up from simple behavioral spaces like moving a cursor on a screen to complex behaviors (Gao and Ganguli, 2015). A clear advantage to the approaches discussed in Section 5 is that we can learn to bring complex vocal behavioral spaces into a compressive low-dimensional behavior spaces, even without a prior model of the structure in that space. For example, Arneodo et al. (2021) find that directly predicting the acoustic structure of zebra finch song from neural data does not perform as well as predicting the parameters of a low-dimensional biophysical model of song production. In the many species in which we do not have access to a biophysical model of vocal production, learned acoustic spaces may be a viable alternative. In contrast, the current state-of-the-art vocal prostheses for speech bypass biophysical models, directly predicting sentences (i.e., symbolic sequences) with the aid of language models (i.e., a sequence model) (Moses et al., 2021). Such methods do not capture important extra-linguistic information such as emotional tone and stress. In future work, a clear pathway forward is to develop BCI models that can both capture symbolic organization aided by sequential models, as well as within-symbol variability in the acoustic signal.

### 8.2. Vocal Production

Songbirds as a model for systems neuroscience are perhaps best known for the role they play in our understanding of vocal learning (Doupe and Kuhl, 1999). In addition to songbirds, rodent and non-human primate vocal behavior are becoming increasingly prominant models in the neuroscience of vocal production. In non-human primates, recent evidence has suggested some degree of constrained vocal learning in some species (Fischer and Hammerschmidt, 2020). In rodents, recent focus has been placed upon variability and structure mouse in ultrasonic vocalizations (USVs) (Holy and Guo, 2005; Arriaga et al., 2012; Petkov and Jarvis, 2012), singing mice have emerged as a physiological model of turn-taking (Okobi et al., 2019), and the cultural transmission of vocal dialect has been observed in the naked mole rat (Barker et al., 2021). In each of these cases, quantification of how vocalizations vary as well as relationships between vocalizations (either within individual, between conspecifics, or from tutor to pupil) is integral to understanding how we learn to navigate vocal space. For example, Kollmorgen et al. (2020) use nearest-neighbor graphs and t-SNE projections to quantify and visualize the developmental trajectory of zebra finch song during vocal learning. For each syllable, they compute a nearest-neighbors graph based metric termed the “neighborhood production time,” which quantifies the developmental time point at which similar (neighboring) syllables were sung. For example, a syllable song on day 45 might have 10 neighbors, sung on days between day 40 and 50, comprising its neighborhood production times. Syllable renditions that are neighbors with predominantly future syllables are deemed anticipations, while syllable repetitions that are neighbors with predominantly past syllables are deemed regressions. They observe that day-by-day, zebra finch songs gradually moves along a constant vocal learning trajectory, but anticipations and regressions differ in how they are consolidated overnight.

**Figure 7:**
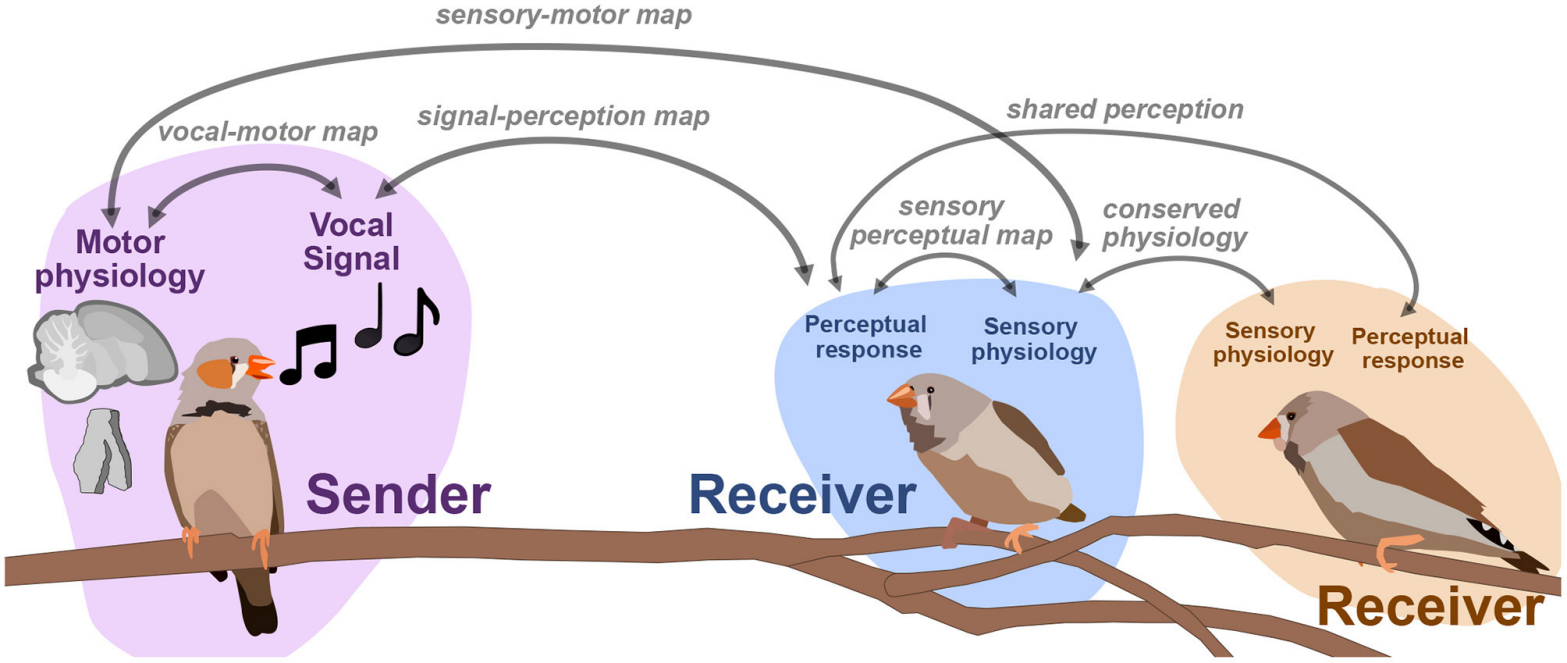
An outline of mappings between perceptual, acoustic, and physiological signals. One focus in sensory and motor neuroscience is to learn the relationships between signals, perception, and physiology.

The number of neurons we can simultaneously record from physiologically has increased the dimensionality of neural datasets substantially over the past decade, making methods for dimensionality reduction on neural signals such as spike trains increasingly necessary for neural data analysis and opening the door to computational methods that directly link the latent representations of behavioral and neural datasets. Population modeling approaches such as LFADS (Latent Factor Analysis via Dynamical Systems; Pandarinath et al., 2018) reduce large population spiking datasets into low-dimensional trajectories, similar to the approaches discussed here with vocal signals. In the case of LFADS, these embeddings are performed over single trials using a recurrent autoencoder. One promising direction for computational neuroethology is learning the relationship between latent behavioral states and latent physiological states. By developing tools that allow us to learn the relationship between physiological and behavioral representations, we hope to untangle how, for example, movements in behavioral space reflect changes in physiology, and vice-versa. Singh Alvarado et al. (2021) developed a joint encoding model in which they used variational autoencoders to learn a joint latent representation of spectrograms of zebra finch song, and corresponding ensemble neural activity of spiny neurons in songbird basal ganglia (average calcium fluorescence of around 60 ROIs, or putative neurons, per bird). In a series of experiments leading up to this joint mapping, Singh Alvarado et al. demonstrated that Area X spiny neurons are involved in the regulation of vocal variability; exhibiting suppressed activity during female-directed song and enhanced activity during practice. Using the joint vocal-neural latent mapping, they were able to uncover the mapping between specific features of song and variants present in neural ensemble activity. In Figure 7 we outline several similar maps between behavior, perception, and neural dynamics. Singh Alvarado et al.'s work exhibit that one such latent map, a vocal-motor mapping between motor physiology and vocal behavior, can uncover complex and detailed relationships that traditional methodology cannot. Similar mappings between the physiology, perception, and behavior of sender-receiver dynamics (e.g., Figure 7) are also well poised to benefit from emerging latent approaches.

The physiology of vocal syntax is another area poised to benefit from computational ethology. One example is the role of the songbird premotor nucleus, HVC, in encoding song syntax. Birdsong has a long history of being described sequentially in terms of low-order Markovian transitions between song elements. HVC's role in song syntax, until recently has been described exclusively in terms of these low-order transition statistics (Fujimoto et al., 2011). In a recent example, however, (Cohen et al., 2020b) made use of an automated birdsong labeling paradigm and high-order sequence model to observe 'hidden neural states' encoding sequentially displaced (i.e., high-order) transitions in the premotor nucleus HVC of canaries. To identify non-adjacent dependencies in the song, they used a Prediction Suffix Tree (Markowitz et al., 2013), which can capture high-order Markovian relationships in the song syntax. Prediction Suffix Trees have previously been used to observe long-range dependencies up to the 7th order in canaries (Markowitz et al., 2013). While birds were singing, Cohen et al., used a miniature microscope to image neurons from HVC, a region involved in the songbird vocal motor circuit. They observed that HVC ROIs were locked to individual song-phrases and transitions, and that this phrase locking is modified by non-adjacent context, displaced by several phrases and seconds. As more recent approaches give access to larger datasets enabling the identification of longer-range dependencies in birdsong, it is currently not clear whether we have yet found an upper bound on the sequential displacement of long-range representations of vocal syntax in physiology.

Outside of songbirds and mammals, male *Drosophila* song, although not strictly vocal, is temporally patterned and driven by both environment and internal states (Coen et al., 2014; Calhoun et al., 2019). Calhoun et al. (2019) jointly model and uncover relationships between temporal song structure and interactions with a potential mate. Using a sequential model (an HMM-GLM hybrid) they demonstrate that song patterning is underlined by three hidden sensorimotor states, under which male's song productions differ in their relationship to female behavior. Using optogenetic activation, they were then able to identify neurons involved in switching between these sensorimotor states.

### 8.3. Vocal Perception

Similar to vocal production, latent and sequential models are promising avenues for better understanding cognitive and physiological underpinnings of vocal perception. In songbirds, primates, and rodents, many foundational studies of auditory categorical perception, perceptual decision making, and their underlying physiology rely upon either relatively simple stimuli such as tones or complex stimuli like human speech (Kuhl and Miller, 1978; Kuhl and Padden, 1983; Russ et al., 2007; ten Cate, 2014; Xin et al., 2019). Categorization in these stimulus spaces are attractive because they are well-characterized and understood. Across species, however, neural responses are often tied to complex and more behaviorally-relevant acoustic phenomena such as recognizing and discriminating between conspecific vocalizations (Bailey et al., 2002; Liu et al., 2019). When the acoustic features underlying vocal repertoires are simple and known, categorical stimuli can be selected directly based upon those features. For example, Lachlan and Nowicki (2015) manipulate a single dimension, the duration of swamp sparrow notes, to determine how notes are categorically perceived in different sequential contexts. In speech, voice onset time (VOT) is a similar single-dimension commonly used for categorical perception paradigms (Liberman et al., 1957). However, it is rarely the case that categorical perception is driven by a single dimension. Thus, building stimuli in more complex feature spaces will be necessary to untangle the relationship between vocal features, perception, and physiology. When biophysical models of vocal structure exist, species relevant stimuli can be generated using biophysical parameters (Arneodo and Mindlin, 2009). When the underlying acoustical structure of a vocal repertoire is more complex and biophysical models of vocalizations have not been defined, neural-network synthesized vocalizations are an attractive alternative. For example, as discussed above, birdsong can be synthesized with neural networks for physiological and perceptual playback studies to determine perceptual similarity between syllables or learn categorical boundaries between song-morphs (Sainburg et al., 2018a; Thielk et al., 2018; Zuidema et al., 2020). By systematically controlling the signal space of a vocal repertoire, we can systematically explore how changes in that space relate to changes in physiology.

Algorithmic approaches are similarly well poised to aid in our understanding of how vocal sequences are maintained and represented. Sequence learning research in human and non-human primates is largely dominated by artificial grammar learning (AGL) research, an umbrella category that comprises several different forms of sequence learning ranging from hierarchically nested tree structures to transitional probabilities (Dehaene et al., 2015). Artificial grammar learning studies aim to determine what structures animals (and humans) are capable of learning, what cognitive mechanisms underlie grammar induction, and what physiological systems underlie those cognitive mechanisms. In the domain of primate sequence learning, neural pathways are generally conserved between humans and non-human primates and involve the ventral regions of cortex (Wilson et al., 2017). Determining an appropriate stimuli set is requisite for developing an AGL paradigm. Latent representations of vocalizations can aid in choosing stimuli from a well-defined stimulus space. For example, when choosing a stimulus set for an *A*^*n*^*B*^*n*^ grammar, it is desirable depending on the goal of the task to ensure that the constituent vocalizations comprising *A* and *B* belong to equidistant or separate clusters in acoustic or perceptual spaces (Zuidema et al., 2020).

While artificial grammar learning has also played a prominent role in birdsong sequence learning (ten Cate and Okanoya, 2012), the structure underlying an animal's own vocal syntax provides an opportunity to study the neural and cognitive underpinnings of a more ethologically-relevant complex sequential structure. Despite the important role vocal syntax production has played in establishing birdsong as a model in systems neuroscience, a surprising gap exists in our knowledge of the physiological circuits underlying how syntactic information is recognized and sequentially integrated when listening to song. Songbird vocal communication contains often very complex syntax that can be structured over long timescales comprising often tens to hundreds of unique, stereotyped vocal units (Cody et al., 2016). Conspecifics pay attention to the structure of that song. Abe and Watanabe (2011) developed a habituation/dishabituation paradigm with Bengalese finches alongside immediate early gene (IEG) expression and lesioning experiments to explore the role of song nuclei on the recognition of grammatical sequences. They found that IEG expression increased when presented with non-conforming/nonpredictive strings in the nuclei LMAN, a basal ganglia output nuclei characterized by recurrent loops that is also involved in vocal learning (Bottjer and Altenau, 2010). Abe and Wantenable then lesioned LMAN and measured song discrimination with their habituation paradigm. They found that discrimination was disturbed in birds where LMAN was lesioned, implicating LMAN in the ability to discriminate syntactic song. How syntactic information is learned, integrated, and maintained in LMAN and associated striatal regions of songbird brain are still open questions.

In contrast to the auditory domain where little is known about syntactic integration, a neural correlate for complex and abstract information integration, NCL, has been well established and characterized in songbird vision with pigeons and corvids (Kröner and Güntürkün, 1999; Güntürkün, 2005). Strong parallels exist between NCL and the primate prefrontal cortex, which is involved in sequence learning. NCL has variously been associated with rule learning (Veit and Nieder, 2013), numerosity (Ditz and Nieder, 2015; Wagener et al., 2018), directed forgetting (Rose and Colombo, 2005; Milmine et al., 2008; Helduser et al., 2013), choice behavior (Kalenscher et al., 2003), working memory (Diekamp et al., 2002; Rinnert et al., 2019), sequence learning (Helduser and Güntürkün, 2012), and reward learning. Anatomically and neurochemically NCL also exhibits strong parallels to the primate prefrontal cortex. NCL is characterized by similar circuitry from auditory and dopaminergic afferents, as well as multi-sensory projections (Kröner and Güntürkün, 1999; von Eugen et al., 2020). Surprisingly, however, an auditory equivalent to the visual working-memory region in NCL has not been found, though they have been observed in the multi-sensory audio-visual integration and association (Moll and Nieder, 2015, 2017). Birdsong is well poised as a signal to be a model of vocal syntax perception, To establish this model, however, it will be imperative to uncover the systems in songbird brain related to working memory and temporal context integration in song. NCL appears to be a likely candidate for processing syntactic vocal signals, though, as yet, this has not been found to be the case.

Although mouse USVs do not appear to contain temporal structure to the same extent as songbirds, mouse USVs are temporally organized (Castellucci et al., 2018) and female mice also show preference for more complex syllables and sequences (Holy and Guo, 2005), making mouse USVs another potential target for the study of syntactic and sequential integration in vocal perception.

## 9. Discussion

This review covers emerging approaches in the computational neuroethology of vocal communication enabling researchers to engage with large and diverse datasets of vocal signals and to represent them in computationally tractable frameworks.

We started by discussing techniques to process and represent acoustic signals. We then discussed how to parse complex vocal datasets into species, individuals, and discrete vocal elements. Next, we discussed how relational structure can be extracted from vocal signals, how these signals can be clustered in learned latent spaces, and how these latent spaces capture different aspects of the information contained within the underlying signals. We then discussed how temporal structure can be inferred from vocal units, including emerging work on the non-Markovian dynamics underlying vocal behavior. In the next section, we discussed how vocalizations can be synthesized for use in playback experiments that allow an unprecedented degree of control over non-linear and complex vocal feature spaces. Finally, we discussed how these approaches are being applied to the field of neuroethology and emerging frameworks for understanding vocal signals and their underlying physiology.

The methods discussed here provide a promising avenue for a broader, more diverse, and larger-scale neuroethology of vocal communication, than the research practices that have dominated the past several decades, and hold the promise of expanding both the breadth and depth of our understanding. Instead of focusing on a small number of model species, new computational techniques provide a framework for studying vocal behavior across a wide range of animals. While much of vocal neuroethology has recently focused on songbirds and mammals the techniques discussed here are equally applicable to the abundance of other species studied in bioacoustics and behavioral ecology including fish, amphibians, and insects. Even within songbirds, research on vocal learning in songbirds has ignored the majority of species, female birdsong, and most call types (Loo and Cain, 2021). Likewise, because these new computational methodologies can often deal with unstructured data, they enable us to expand beyond simplified, isolated behaviors in controlled environments to more natural or naturalistic behavioral contexts where dynamics involving multi-modal integration and multi-animal social interactions arise. As we capture increasing levels of detail in behavior, our understanding of its sophistication naturally follows. Already, these new computational framework have revealed deep structure in the sequential organization of communication, where large-scale datasets of both symbolic sequences, and latent projections that capture rendition-to-rendition variability, have enabled quantitative analyses of rare (but perhaps meaningful) events, such as long-range syntactic organization. Together, all of these approaches point toward a new framework, in which complex and non-linear behavioral and physiological signals can be represented in compressive and tractable spaces that can capture the complex dynamics and relationships in the increasingly rich datasets available to researchers.

As with any powerful tool, these techniques require careful consideration when put into practice. Broadly, automation and machine learning in data analysis can be fraught with unexpected complications and confounds that may be hard to spot. For example, automating the labeling of large datasets of birdsong syllables can speed up the task of labeling by days, weeks, or months, but can also leave the experimenter with less intuitive knowledge of the animal's vocal repertoire, resulting in a loss of domain knowledge. As we have noted elsewhere (Sainburg et al., 2020b), when domain knowledge is available it should be integrated with one computational approach. Another potential pitfall (and a source of much needed research effort) is in understanding the structure of the latent manifolds that are yielded in many of the described methods. In particular, non-linear latent modeling techniques like UMAP or neural networks can capture complex relationships in vocal data, but interpreting these projections requires an understanding of how data are represented within the geometry of the latent space. For example, UMAP captures primarily local structure in datasets that are present in nearest neighbor graphs, meaning that the relative distances of vocal elements have no explicit relation to the data, as is the case in PCA for example.

Attending to the cautions of computational abstraction, the approaches discussed in this manuscript provide a framework from which to quantify vocal signals that promises to yield important new insights into vocal behavior and neurobiology. These approaches enable neuroethologists to project vocalizations onto low dimensionaland latent manifolds, visualize and quantify the transitional structure and information decay of vocal syntax, and map vocal and neural repertoires into shared neural spaces for functional representation action. As the richness of datasets grow to capture more of the complexities of behavior and physiology, methods and frameworks for modeling and inferring structure in ethological data are increasingly necessary for hypothesis formulation and testing. The methods and frameworks discussed in this review parallel and supplement those in the broader field of computational neuroethology.

## Author Contributions

This manuscript was written by TS and TG. Both authors contributed to the article and approved the submitted version.

## Funding

This work was supported by a CARTA Fellowship to TS, NIH 5T32MH020002-20 to TS, and 5R01DC018055-02 to TG.

## Conflict of Interest

The authors declare that the research was conducted in the absence of any commercial or financial relationships that could be construed as a potential conflict of interest.

## Publisher's Note

All claims expressed in this article are solely those of the authors and do not necessarily represent those of their affiliated organizations, or those of the publisher, the editors and the reviewers. Any product that may be evaluated in this article, or claim that may be made by its manufacturer, is not guaranteed or endorsed by the publisher.
